# Cardiopulmonary resuscitation knowledge and skills among school teachers in Xinjiang, China: a cross-sectional survey

**DOI:** 10.3389/fpubh.2025.1683122

**Published:** 2025-10-16

**Authors:** Jin Ma, Liuniu Kuai, Xiaolong Zhu, Qi Tang, Shifang Liu, Weiwei Zhou

**Affiliations:** ^1^Department of Emergency Medicine, The People’s Hospital of Atushi City, Atushi, China; ^2^Department of Urology Surgery, The People’s Hospital of Atushi City, Atushi, China; ^3^Department of Cardiovascular Medicine, The People’s Hospital of Atushi City, Atushi, China

**Keywords:** cardiopulmonary resuscitation, school teachers, emergency preparedness, CPR training, Xinjiang, China

## Abstract

**Background:**

Cardiopulmonary resuscitation (CPR) is a critical, life-saving intervention that is especially important in school settings. This study assessed the levels of CPR knowledge, training, and rescue willingness among school teachers in Xinjiang, China.

**Methods:**

We conducted an online cross-sectional survey among 368 full-time teachers across primary, middle, and high schools (May–June 2025). A composite CPR readiness score (0–100) combined self-reported knowledge, training (formal or self-study vs. none), four core technical items (golden time, compression location, depth, rate), and rescue willingness. Group differences were assessed by Kruskal–Wallis with FDR-adjusted Mann–Whitney *post hoc* tests; categorical associations used chi-square with Cramer’s V; multivariable correlates of readiness were examined by OLS with robust SEs.

**Results:**

Overall, 37.0% were trained (self-study or formal); 10.9% reported “very clear” knowledge. Technical knowledge accuracy was uneven: compression location 78.0%, golden time within 4 min 68.5%, compression rate 100–120/min 44.8%, and depth 5–6 cm 39.7%. Mean readiness was 55.7 ± 17.4 (95% CI: 53.9–57.4) and was higher in trained than untrained teachers (69.8 ± 13.3 vs. 47.4 ± 13.8; *p* < 0.0001). Readiness differed by age (*p* = 0.019; lower in 46–60 years) and ethnicity (Han 57.2 ± 16.6 vs. other minorities 52.8 ± 18.5; *p* = 0.027), and was higher among those aware of AED locations (69.3 ± 16.0; *p* < 0.0001). In OLS, training (+20.39 points [95% CI: 17.54–23.23]; *p* < 0.0001), AED awareness (+8.35 [4.09–12.62]; *p* = 0.000124), and often worrying about emergencies (+12.09 [4.37–19.81]; *p* = 0.002) were independent positive correlates; male sex (−3.54 [−6.91 to −0.18]; *p* = 0.039) and other minority ethnicity (−5.57 [−8.99 to −2.14]; *p* = 0.001) were negative.

**Conclusion:**

The findings demonstrate an urgent need for systematic and culturally adapted CPR training programs among school teachers in Xinjiang, China.

## Introduction

Out-of-hospital cardiac arrest (OHCA) remains one of the leading causes of mortality worldwide, with survival being heavily dependent on immediate intervention (1–3). The concept of the “chain of survival” indicates that early recognition, prompt initiation of cardiopulmonary resuscitation (CPR), and rapid defibrillation within the first few minutes can significantly increase survival probabilities from less than 10% to over 50% (4–10). In school environments where professional medical assistance is rarely immediately available, teachers are unlikely to have more than a few minutes to respond to life-threatening emergencies, making their ability to perform CPR essential. In China, where student-to-teacher ratios are high and school hours are long, the likelihood of witnessing a cardiac emergency is non-negligible.

International studies have documented wide variability in teacher CPR preparedness. While CPR training rates in high-income countries with mandatory training policies can exceed 50%, reports from other regions suggest substantially lower rates (11–17). In the ethnically and culturally diverse region of Xinjiang, China, unique challenges such as geographical isolation, limited access to quality healthcare training, and language barriers may further impede the acquisition and retention of CPR skills.

Against this backdrop, we conducted a cross-sectional survey to characterize CPR knowledge, training experience, and overall readiness among school teachers in Xinjiang. We further examined differences across demographic and contextual subgroups, mapped associations among key categorical factors, and identified multivariable correlates of CPR readiness to inform targeted, scalable interventions. Unlike many prior studies, we operationalized readiness as a composite construct integrating knowledge, training, core technical accuracy, and willingness—thus capturing preparedness more holistically than any single domain alone.

## Methods

### Study design and participants

We administered an online, anonymous, cross-sectional survey to full-time teachers across primary, middle, and high schools in Atushi, Xinjiang province, China from May to June 2025. The survey was distributed through WeChat groups associated with regional teacher associations. Eligibility criteria included active classroom teaching, direct student contact, Chinese literacy, and informed consent. Of 373 responses, 368 were retained after data cleaning and completeness checks.

### Measures

The survey instrument was developed based on validated international CPR knowledge assessments and adapted for the Chinese context. The questionnaire underwent translation and back-translation by independent bilingual team members to ensure conceptual equivalence. English translations of all key survey items are provided in Supplementary Methods. The questionnaire comprised five sections. Demographics comprised gender, age, ethnicity (analyzed as Han vs. other minorities), education, and teaching level (primary, middle, and high). CPR knowledge and awareness included self-reported knowledge level, recognition of appropriate indications for CPR, awareness of school automated external defibrillator (AED) locations, and related perceptions. Training experience captured any prior training (self-study or formal), time since last training, and self-assessed skill mastery among those trained. Technical knowledge items assessed golden rescue time, chest compression location, depth, rate, and AED use timing. Emergency-related attitudes encompassed worry about emergencies, rescue willingness, volunteer willingness, and perceived necessity of teacher CPR training.

### CPR readiness score

The primary outcome was a composite CPR readiness score (0–100) defined *a priori* from four domains: (1) self-reported CPR knowledge (0–30); (2) training experience (0–20: formal 20, self-study 10, none 0); (3) technical accuracy on four core items (0–30: golden time, compression location, depth, rate; 7.5 points each if correct); and (4) rescue willingness (0–20: willing & competent 20; willing but worried 15; need legal protection 10; not willing 0). The weighting scheme was developed through expert consensus involving three emergency physicians, aligning with international CPR assessment frameworks. Internal consistency was assessed using Cronbach’s alpha. To test robustness, we conducted sensitivity analyses with ±20% variations in component weights.

### Statistical analysis

We summarized categorical variables as n (%) and continuous variables as mean ± SD with 95% confidence intervals (CI). Baseline characteristics were described overall and by training status (untrained vs. trained). Group differences for readiness used Kruskal–Wallis tests with Mann–Whitney *post hoc* comparisons when overall *p* < 0.05. Effect sizes were calculated using Cramer’s *V* for categorical associations, with values of 0.1, 0.3, and 0.5 representing small, medium, and large effects, respectively. To identify independent correlates of readiness, we fitted ordinary least squares (OLS) models with robust (HC3) standard errors; predictors included training (any vs. none), gender, age group, ethnicity, education, teaching level, AED location awareness, and worry about emergencies. Model assumptions were verified through comprehensive residual diagnostics including Q-Q plots, residuals versus fitted values plots, and scale-location plots. Multicollinearity was assessed using variance inflation factors (VIF), with values exceeding 5.0 considered problematic. All regression coefficients are reported with 95% confidence intervals. Statistical significance was set at *p* < 0.05.

## Results

### Participant characteristics

Among 368 teachers, 280 (76.1%) were female and 88 (23.9%) male (Table 1). Age distribution was 18–25 years 11.7%, 26–35 years 45.7%, 36–45 years 32.9%, and 46–60 years 9.8%. Ethnicity was Han 65.2% and other minorities 34.8%. Most held a bachelor’s degree (91.0%). Teaching levels were primary 54.1%, high 30.4%, and middle 15.5%. Overall, 37.0% were trained (self-study or formal).

**Table 1:** The demographic information of the participants and the characteristics of the questionnaire (*N* = 368).

Variable	Overall	Untrained	Trained	*p*-value	Cramer’s *V*
Gender				0.03183	0.11
Female	280 (76.1%)	185 (79.7%)	95 (69.9%)		
Male	88 (23.9%)	47 (20.3%)	41 (30.1%)		
Age				0.04135	0.15
18–25 years	43 (11.7%)	22 (9.5%)	21 (15.4%)		
26–35 years	168 (45.7%)	109 (47.0%)	59 (43.4%)		
36–45 years	121 (32.9%)	72 (31.0%)	49 (36.0%)		
46–60 years	36 (9.8%)	29 (12.5%)	7 (5.1%)		
Ethnicity				0.8747	
Han	240 (65.2%)	152 (65.5%)	88 (64.7%)		
Other minorities	128 (34.8%)	80 (34.5%)	48 (35.3%)		
Education				0.3894	
Bachelor	335 (91.0%)	210 (90.5%)	125 (91.9%)		
Junior college	28 (7.6%)	17 (7.3%)	11 (8.1%)		
Postgraduate	3 (0.8%)	3 (1.3%)	0 (0.0%)		
Technical secondary	2 (0.5%)	2 (0.9%)	0 (0.0%)		
Teaching level				0.000115	0.22
High	112 (30.4%)	59 (25.4%)	53 (39.0%)		
Middle	57 (15.5%)	28 (12.1%)	29 (21.3%)		
Primary	199 (54.1%)	145 (62.5%)	54 (39.7%)		
Readiness score	55.7 ± 17.4	47.4 ± 13.8	69.8 ± 13.3	3.02E-35	

### CPR knowledge and awareness

Self-reported CPR knowledge levels revealed concerning gaps: only 10.9% of teachers reported “very clear” knowledge enabling complete procedure description, while 69.6% had “general understanding but unclear details,” 15.5% had “only heard of the concept,” and 4.1% reported “no knowledge” (Table 1). Recognition of appropriate CPR application scenarios was moderate, with 57.1% correctly identifying “unresponsive with absent or abnormal breathing” as requiring immediate CPR. Awareness of AED device locations was limited, with only 17.7% knowing their school’s AED locations.

### Training experience and skill retention

Overall CPR training rate was 37.0%, comprising 10.9% formal training through hospitals or Red Cross organizations and 26.8% self-study through videos or books (Supplementary Table 1). Among trained individuals (*n* = 136), training recency varied: within 6 months (29.4%), 6–12 months (25.0%), 1–2 years (16.2%), and over 2 years (29.4%). Skill retention among trained personnel was suboptimal: 12.5% felt capable of independent operation, 61.8% remembered steps but felt rusty, 21.3% retained only theoretical knowledge, and 4.4% had almost forgotten their training. Training status varied by gender (*p* = 0.032, Cramer’s *V* = 0.11), age (*p* = 0.041, Cramer’s *V* = 0.15), and teaching level (*p* < 0.001, Cramer’s *V* = 0.22), but not by ethnicity or education level.

### CPR technical knowledge accuracy

Technical CPR knowledge was unevenly distributed among the respondents: 78.0% correctly identified the center of the chest as the optimal compression site, 68.5% recognized the recommended “golden time” interval of approximately 4 min for effective resuscitation, yet only 44.8 and 39.7% correctly reported the recommended compression rate (100–120/min) and depth (5–6 cm), respectively (Supplementary Table 1). On average, respondents answered 3.26 ± 1.52 of the six key technical questions correctly, with only 8.6% answering all items accurately and 23.9% responding correctly to two or fewer items.

### Rescue willingness and barriers

While 78.2% of teachers expressed willingness to perform CPR, confidence levels varied significantly: 27.7% felt “willing and capable,” 50.5% were “willing but worried about mistakes,” 17.4% would act only with “legal protection,” and 4.3% were “unwilling to participate” (Supplementary Table 1). Concerns about encountering CPR situations were prevalent: 23.9% “frequently worried,” 47.6% “occasionally worried,” 21.7% “rarely worried,” and 6.8% “never worried.” Legal concerns emerged as a significant barrier, with 17.4% requiring legal protection before performing CPR.

### Training preferences and perceived needs

The most preferred training methods were regular hands-on practice sessions (69.3%), expert lectures with certification (53.0%), and online courses with simulation (49.5%). Willingness to join CPR promotion teams was moderate: 48.1% willing, 36.1% depending on schedule, and 15.8% not considering (Supplementary Table 1). Notably, 83.7% of participants rated CPR training for teachers as “necessary” or “very necessary,” with only 3.0% considering it “unnecessary.” This high perceived need contrasted sharply with the low actual training rate.

### The correlation between CPR training and CPR readiness

Training status was associated with multiple dimensions of preparedness in baseline comparisons. Trained teachers were more likely to report “very clear” CPR knowledge (21.3% vs. 4.7%) and less likely to report “only heard” (4.4% vs. 22.0%) or “no knowledge” (1.5% vs. 5.6%; *p* < 0.0001, Cramer’s *V* = 0.34) (Supplementary Table 1). They more often knew the location of AEDs (Yes: 33.1% vs. 8.6%; Not sure: 33.1% vs. 41.8%; No: 33.8% vs. 49.6%; *p* < 0.0001, Cramer’s *V* = 0.31). Technical knowledge distributions also favored trained teachers: correct chest compression location (88.2% vs. 72.0%; *p* = 0.000136, Cramer’s *V* = 0.24), correct depth 5–6 cm (54.4% vs. 31.0%) with far fewer “not sure” responses (2.2% vs. 26.7%; *p* < 0.0001, Cramer’s *V* = 0.34), correct rate 100–120/min (52.9% vs. 40.1%) with markedly fewer “not sure” (1.5% vs. 23.7%; *p* < 0.0001, Cramer’s *V* = 0.30), and golden time within 4 min (74.3% vs. 65.1%; *p* = 0.0045, Cramer’s *V* = 0.19). Recognition of correct CPR indications was higher among the trained (“unresponsive and not breathing (agonal)”: 67.6% vs. 50.9%; p < 0.0001, Cramer’s *V* = 0.25). Volunteer willingness also differed (willing: 55.9% vs. 43.5%; not now: 9.6% vs. 19.4%; *p* = 0.017, Cramer’s *V* = 0.15). Training status varied modestly by gender and age distributions (*p* = 0.0318 and *p* = 0.0413, respectively), whereas education and ethnicity did not.

### CPR readiness score

**Figure 1:**
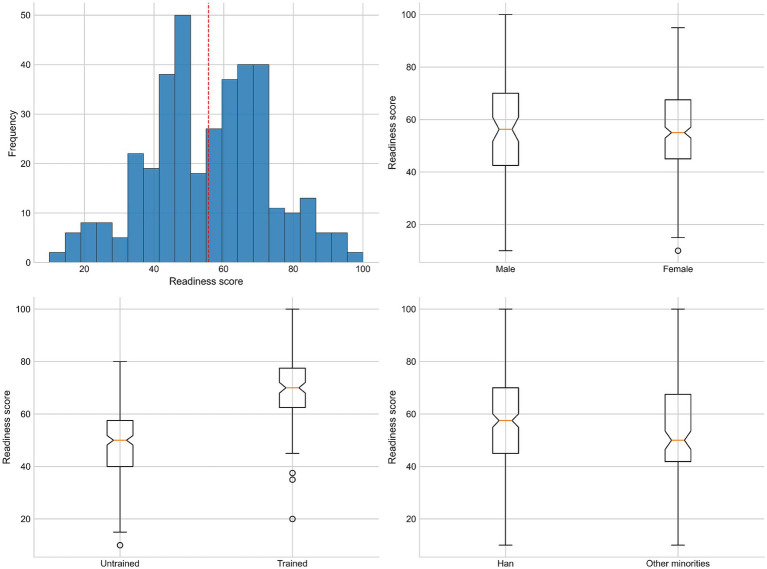
Statistical analysis of CPR readiness scores. Overall distribution (histogram) and group comparisons by gender, training status, and ethnicity.

Mean readiness was 55.7 ± 17.4 (95% CI: 53.9–57.4) (Figure 1). By training status, trained teachers scored higher than untrained (69.8 ± 13.3 [67.5–72.0] vs. 47.4 ± 13.8 [45.6–49.2]; Kruskal–Wallis *p* < 0.0001) (Table 2). Readiness did not differ by gender (*p* = 0.954) or education (*p* = 0.750) and showed a non-significant trend across teaching levels (*p* = 0.061). Age differences were significant overall (*p* = 0.0188); FDR-adjusted pairwise tests indicated lower readiness among 46–60 years compared with 18–25 years (*p* = 0.0339) and 36–45 years (*p* = 0.0339), with other contrasts not significant. Ethnicity was associated with readiness (Han 57.2 ± 16.6 vs. other minorities 52.8 ± 18.5; *p* = 0.0273; pairwise *p* = 0.0274).

**Table 2:** The differences in CPR readiness scores among different subgroups.

Group variable	Level	*N*	Mean ± SD	Median [IQR]	Test	Statistic	*p*-value
Gender	Female	280	55.8 ± 17.0	55.0 [45.0, 67.5]			
Gender	Male	88	55.1 ± 18.6	56.2 [42.5, 70.0]			
Gender	Overall test	368			Kruskal–Wallis	0.003376	0.953669
Age	26–35 years	168	54.4 ± 16.1	52.5 [42.5, 67.5]			
Age	36–45 years	121	57.6 ± 18.5	57.5 [47.5, 70.0]			
Age	18–25 years	43	60.8 ± 18.0	57.5 [50.0, 71.2]			
Age	46–60 years	36	49.0 ± 16.6	50.0 [37.5, 63.1]			
Age	Overall test	368			Kruskal–Wallis	9.976932	0.018763
Ethnicity	Han	240	57.2 ± 16.6	57.5 [45.0, 70.0]			
Ethnicity	Other minorities	128	52.8 ± 18.5	50.0 [41.9, 67.5]			
Ethnicity	Overall test	368			Kruskal–Wallis	4.868789	0.027347
Education	Bachelor	335	55.6 ± 17.4	55.0 [42.5, 67.5]			
Education	Junior college	28	57.8 ± 17.3	53.8 [47.5, 65.0]			
Education	Postgraduate	3	48.3 ± 21.3	47.5 [37.5, 58.8]			
Education	Technical secondary	2	47.5 ± 14.1	47.5 [42.5, 52.5]			
Education	Overall test	368			Kruskal–Wallis	1.214189	0.749603
Teaching level	Primary	199	53.7 ± 16.0	52.5 [42.5, 65.0]			
Teaching level	High	112	57.7 ± 18.8	57.5 [46.9, 70.0]			
Teaching level	Middle	57	58.5 ± 18.4	60.0 [45.0, 72.5]			
Teaching level	Overall test	368			Kruskal–Wallis	5.605114	0.060655
CPR knowledge	General	256	57.6 ± 13.2	57.5 [50.0, 67.5]			
CPR knowledge	Only heard	57	39.4 ± 12.6	40.0 [32.5, 45.0]			
CPR knowledge	Very clear	40	77.5 ± 13.1	78.8 [70.0, 87.5]			
CPR knowledge	No knowledge	15	25.7 ± 12.8	20.0 [16.2, 36.2]			
CPR knowledge	Overall test	368			Kruskal–Wallis	143.6237	6.25E-31
Know AED location	No	161	52.9 ± 15.6	52.5 [42.5, 65.0]			
Know AED location	Not sure	142	52.6 ± 17.0	52.5 [42.5, 67.5]			
Know AED location	Yes	65	69.3 ± 16.0	70.0 [60.0, 82.5]			
Know AED location	Overall test	368			Kruskal–Wallis	44.68428	1.98E-10
When CPR is needed	Unresponsive and not breathing (agonal)	210	61.0 ± 15.7	62.5 [50.0, 70.0]			
When CPR is needed	Pulse present but dyspnea	106	53.4 ± 15.6	52.5 [42.5, 64.4]			
When CPR is needed	Not sure	45	37.8 ± 15.3	40.0 [25.0, 47.5]			
When CPR is needed	Conscious with chest pain	7	46.1 ± 16.8	40.0 [37.5, 55.0]			
When CPR is needed	Overall test	368			Kruskal–Wallis	64.82352	5.47E-14
Golden time	Within 4 min	252	60.3 ± 15.6	60.0 [50.0, 70.0]			
Golden time	Within 8 min	57	50.5 ± 14.1	50.0 [42.5, 60.0]			
Golden time	Not sure	44	35.6 ± 14.5	35.0 [25.0, 42.5]			
Golden time	Over 10 min	15	56.3 ± 18.0	52.5 [43.8, 66.2]			
Golden time	Overall test	368			Kruskal–Wallis	73.82657	6.47E-16
Compression location	Chest center	287	60.2 ± 15.2	60.0 [50.0, 70.0]			
Compression location	Left chest	45	46.0 ± 14.6	45.0 [37.5, 52.5]			
Compression location	Not sure	28	29.2 ± 11.0	30.0 [20.0, 35.0]			
Compression location	Right chest	8	41.6 ± 12.7	41.2 [35.0, 48.8]			
Compression location	Overall test	368			Kruskal–Wallis	96.49295	8.82E-21
Compression depth	5–6 cm	146	66.7 ± 13.3	67.5 [60.0, 72.5]			
Compression depth	3–4 cm	145	53.2 ± 14.2	52.5 [45.0, 62.5]			
Compression depth	Not sure	65	36.3 ± 12.2	40.0 [27.5, 45.0]			
Compression depth	>6 cm	12	55.4 ± 17.2	57.5 [37.5, 70.6]			
Compression depth	Overall test	368			Kruskal–Wallis	146.4253	1.56E-31
Compression rate	100-120/min	165	66.1 ± 13.7	65.0 [57.5, 75.0]			
Compression rate	60-100/min	138	52.4 ± 13.2	50.0 [42.5, 62.5]			
Compression rate	Not sure	57	34.3 ± 12.1	35.0 [25.0, 42.5]			
Compression rate	>120/min	8	50.3 ± 18.5	53.8 [35.6, 65.6]			
Compression rate	Overall test	368			Kruskal–Wallis	148.7474	4.91E-32
AED timing	Immediate analysis and shock	147	59.0 ± 15.8	60.0 [50.0, 70.0]			
AED timing	Use after 2 min CPR	121	61.7 ± 15.9	62.5 [50.0, 70.0]			
AED timing	Not sure	100	43.5 ± 15.2	42.5 [35.0, 50.6]			
AED timing	Overall test	368			Kruskal–Wallis	70.33529	5.33E-16
Worry about emergencies	Sometimes	175	56.3 ± 16.7	57.5 [45.0, 68.8]			
Worry about emergencies	Often	88	57.7 ± 17.0	60.0 [45.0, 70.0]			
Worry about emergencies	Seldom	80	54.4 ± 17.1	52.5 [42.5, 65.0]			
Worry about emergencies	Never	25	48.0 ± 22.5	50.0 [35.0, 55.0]			
Worry about emergencies	Overall test	368			Kruskal–Wallis	6.370029	0.094932
Rescue willingness	Willing but worried	186	56.1 ± 15.8	56.2 [43.1, 67.5]			
Rescue willingness	Willing & competent	102	62.8 ± 16.3	62.5 [50.6, 72.5]			
Rescue willingness	Need legal protection	64	48.9 ± 17.0	52.5 [37.5, 60.6]			
Rescue willingness	Not willing	16	32.0 ± 13.6	31.2 [20.0, 43.1]			
Rescue willingness	Overall test	368			Kruskal–Wallis	46.38975	4.69E-10
Skill mastery after training	Remember steps but rusty	84	71.1 ± 10.5	70.0 [66.9, 75.0]			
Skill mastery after training	Know theory only	29	63.4 ± 13.4	62.5 [55.0, 70.0]			
Skill mastery after training	Competent independently	17	81.5 ± 11.4	82.5 [72.5, 90.0]			
Skill mastery after training	Almost forgotten	6	48.3 ± 15.9	51.2 [45.6, 55.0]			
Skill mastery after training	Overall test	136			Kruskal–Wallis	30.41887	1.13E-06
Time since last training	≤6 months	40	75.6 ± 16.5	78.8 [67.5, 85.6]			
Time since last training	>2 years	40	64.6 ± 9.6	67.5 [57.5, 70.0]			
Time since last training	6–12 months	34	71.5 ± 9.2	70.0 [67.5, 75.0]			
Time since last training	1–2 years	22	66.0 ± 14.0	68.8 [58.1, 74.4]			
Time since last training	Overall test	136			Kruskal–Wallis	20.78054	0.000117
Volunteer willingness	Willing	177	60.0 ± 15.8	62.5 [50.0, 70.0]			
Volunteer willingness	Depends on time	133	55.7 ± 15.8	52.5 [45.0, 67.5]			
Volunteer willingness	Not now	58	42.3 ± 18.8	42.5 [25.0, 54.4]			
Volunteer willingness	Overall test	368			Kruskal–Wallis	36.62026	1.12E-08
Training necessity	Very necessary	198	58.8 ± 17.1	60.0 [47.5, 70.0]			
Training necessity	Necessary	110	56.2 ± 15.3	56.2 [45.6, 67.5]			
Training necessity	Average	42	47.4 ± 15.3	47.5 [35.0, 57.5]			
Training necessity	Not necessary	11	32.7 ± 21.0	20.0 [18.8, 47.5]			
Training necessity	Not very necessary	7	44.6 ± 18.5	40.0 [35.0, 53.8]			
Training necessity	Overall test	368			Kruskal–Wallis	29.30044	6.79E-06

### Multivariable correlates of CPR readiness

In the OLS model with robust (HC3) standard errors (adjusted *R*^2^ = 0.49), independent positive correlates of readiness were training (any vs. none: +20.39 points [95% CI: 17.54–23.23], standardized *β* = 0.57; *p* < 0.0001), AED location awareness (Yes vs. No: +8.35 [4.09–12.62], standardized *β* = 0.18; *p* = 0.000124), and often worrying about emergencies (Yes vs. never: +12.09 [4.37–19.81], standardized *β* = 0.30; *p* = 0.00215). Male gender (−3.54 [REVISED] [−6.91 to −0.18], standardized *β* = −0.09; *p* = 0.039) and other minority ethnicity (−5.57 [−8.99 to −2.14], standardized *β* = −0.15; *p* = 0.00145) were independently associated with lower readiness (Table 3 and Supplementary Figure 1). Age group, education, teaching level, and being “not sure” about AED location were not significant after adjustment. Variance inflation factors ranged from 1.03 to 4.39, all below the threshold of 5.0, indicating acceptable levels of multicollinearity. Stratified analyses showed that the positive effect of AED awareness persisted even among untrained teachers (+8.63 points, *p* = 0.023), suggesting independent contributions beyond training effects.

**Table 3:** Independent predictors associated with CPR readiness using OLS models.

Predictor	Coefficient	Std. error	*t*-value	*p*-value	95% CI Lower	95% CI Upper	Standardized Coef	VIF
Intercept	44.155	3.645	12.115	0.000	37.012	51.299	–	–
C (Gender) [T. Male]	−3.544	1.719	−2.062	0.039	−6.912	−0.175	−0.087	1.107
C (Age) [T.26–35 years]	−1.394	2.272	−0.614	0.539	−5.848	3.060	−0.040	3.133
C (Age) [T.36–45 years]	0.195	2.316	0.084	0.933	−4.344	4.735	0.005	2.863
C (Age) [T.46–60 years]	−4.592	3.282	−1.399	0.162	−11.024	1.839	−0.079	1.900
C (Ethnicity) [T. Other minorities]	−5.568	1.748	−3.186	0.001	−8.993	−2.142	−0.153	1.299
C (Education) [T. Junior college]	2.936	2.425	1.210	0.226	−1.818	7.690	0.045	1.086
C (Education) [T. Postgraduate]	−0.026	13.668	−0.002	0.998	−26.814	26.762	0.000	1.033
C (Education) [T. Technical secondary]	−1.533	17.721	−0.087	0.931	−36.266	33.199	−0.006	1.048
C (Teaching Level) [T. Middle]	−2.447	2.217	−1.104	0.270	−6.793	1.899	−0.051	1.419
C (Teaching Level) [T. Primary]	−2.087	1.796	−1.162	0.245	−5.607	1.433	−0.060	1.733
C (AED Location) [T. Not sure]	−0.023	1.518	−0.015	0.988	−2.998	2.952	−0.001	1.198
C (AED Location) [T. Yes]	8.352	2.176	3.839	0.000	4.088	12.617	0.184	1.336
C (Worry Emergency) [T. Often]	12.090	3.940	3.069	0.002	4.368	19.812	0.297	3.666
C (Worry Emergency) [T. Seldom]	6.225	3.974	1.566	0.117	−1.564	14.013	0.148	3.450
C (Worry Emergency) [T. Sometimes]	7.075	3.768	1.878	0.060	−0.310	14.460	0.204	4.385
Trained any	20.386	1.453	14.032	0.000	17.538	23.233	0.567	1.187

## Discussion

This study provides a comprehensive, contemporary appraisal of CPR preparedness among school teachers in Xinjiang, China. Overall readiness was modest (55.7 ± 17.4), and only 37.0% of teachers had ever received CPR training, underscoring a critical implementation gap in a setting where teachers may be the first or only responders during the initial minutes of cardiac arrest. Although nearly 78% of educators expressed willingness to perform CPR, only a fraction felt capable when confronted with an emergency, a discrepancy that is likely to have significant implications for outcomes during actual cardiac arrest events. Against the backdrop of the international “chain of survival” concept, which demonstrates that early bystander CPR and defibrillation can dramatically improve survival rates, our findings suggest that even modest improvements in training coverage and skill sustainability could yield measurable benefits in school settings (4–10, 18, 19).

The comparison with international standards shows that the current training rate of 37.3% among teachers in Xinjiang, China is considerably lower than that reported in regions with mandatory training policies (17, 20–23). The uneven performance on technical questions is particularly disconcerting given that incorrect knowledge regarding compression rate and depth may detract from the overall quality of resuscitative efforts. Cultural factors such as language barriers and differing perceptions of legal responsibility for bystander intervention may contribute to both the low training uptake and the reluctance to act, despite high willingness levels.

The observed differences in readiness scores between Han teachers and those from other minority groups (57.2 ± 16.6 vs. 52.8 ± 18.5, *p* = 0.027) warrant careful interpretation within the complex socio-cultural context of Xinjiang. These disparities should not be interpreted as reflecting inherent differences between ethnic groups, but rather as indicators of systemic inequities in access to resources and opportunities for professional development. The independent negative association of minority ethnicity with readiness in multivariable analysis (*β* = −0.15, *p* = 0.001) persisted after controlling for other factors, suggesting that unmeasured structural barriers contribute to these disparities.

Our composite readiness score showed modest internal consistency (Cronbach’s *α* = 0.44), which is below the conventional threshold of 0.70. This likely reflects the multidimensional nature of CPR readiness, encompassing distinct domains of knowledge, training, technical skills, and psychological willingness that may not form a unidimensional construct. In addition, legal concerns emerged as a significant systemic barrier, with 17.4% of teachers requiring legal protection before being willing to perform CPR. This finding reflects broader societal concerns in China about legal liability for good-faith emergency interventions.

While the study was limited by its cross-sectional design, reliance on self-reported measures (which may overestimate actual CPR competency), and the single geographic region from which the questionnaire sample was drawn, it nonetheless provides critical insights that are directly applicable to the design of future educational interventions. Our data strongly advocate for the institution of mandatory, standardized CPR training programs within the educational system. With the proper deployment of training, AED infrastructure, and legal safeguards, teachers can be empowered to serve as confident and capable first responders, ultimately ensuring greater safety for students and the broader community.

## Data Availability

The original contributions presented in the study are included in the article/Supplementary material, further inquiries can be directed to the corresponding author.
