# Supplementary material for: Cardiopulmonary resuscitation knowledge and skills among school teachers in Xinjiang, China: a cross-sectional survey
Source: Front Public Health. 2025 Oct 16;13:1683122. doi: 10.3389/fpubh.2025.1683122 (PMC12571768; doi:10.3389/fpubh.2025.1683122)
Supplement: Supplementary file 1 [file Supplementary_file_1.docx]

Jin Ma^1#^, Liuniu Kuai^2#^, Xiaolong Zhu^1^, Qi Tang^1^, Shifang Liu^1^ and Weiwei Zhou^3^*

^1^Department of Emergency Medicine, the People’s Hospital of Atushi City,Atushi,China,845300.

^2^Department of Urology Surgery, the People’s Hospital of Atushi City,Atushi,China,845300.

^3^Department of Cardiovascular Medicine, the People’s Hospital of Atushi City,Atushi,China,845300.

***Correspondence:**

Weiwei Zhou,Dr.,Email:weigoo1224@163.com,Department of Cardiovascular Medicine, the People’s Hospital of Atushi City ,No. 6 Guangming North Road,Atushi ,845300 ,China.

^#^ These authors contributed equally to this work.

**Supplementary Table 1. The demographic information of the participants and the characteristics of the questionnaire (N = 368).**

| **Variable** | **Category** | **Overall** | **Untrained** | **Trained** | **Test** | **p-value** |
| --- | --- | --- | --- | --- | --- | --- |
| Gender | Female | 280 (76.1%) | 185 (79.7%) | 95 (69.9%) | Chi-square | 0.031828 |
| Gender | Male | 88 (23.9%) | 47 (20.3%) | 41 (30.1%) | Chi-square | 0.031828 |
| Age | 18-25 years | 43 (11.7%) | 22 (9.5%) | 21 (15.4%) | Chi-square | 0.041347 |
| Age | 26-35 years | 168 (45.7%) | 109 (47.0%) | 59 (43.4%) | Chi-square | 0.041347 |
| Age | 36-45 years | 121 (32.9%) | 72 (31.0%) | 49 (36.0%) | Chi-square | 0.041347 |
| Age | 46-60 years | 36 (9.8%) | 29 (12.5%) | 7 (5.1%) | Chi-square | 0.041347 |
| Ethnicity | Han | 240 (65.2%) | 152 (65.5%) | 88 (64.7%) | Chi-square | 0.874662 |
| Ethnicity | Other minorities | 128 (34.8%) | 80 (34.5%) | 48 (35.3%) | Chi-square | 0.874662 |
| Education | Bachelor | 335 (91.0%) | 210 (90.5%) | 125 (91.9%) | Chi-square | 0.389387 |
| Education | Junior college | 28 (7.6%) | 17 (7.3%) | 11 (8.1%) | Chi-square | 0.389387 |
| Education | Postgraduate | 3 (0.8%) | 3 (1.3%) | 0 (0.0%) | Chi-square | 0.389387 |
| Education | Technical secondary | 2 (0.5%) | 2 (0.9%) | 0 (0.0%) | Chi-square | 0.389387 |
| Teaching level | High | 112 (30.4%) | 59 (25.4%) | 53 (39.0%) | Chi-square | 0.000115 |
| Teaching level | Middle | 57 (15.5%) | 28 (12.1%) | 29 (21.3%) | Chi-square | 0.000115 |
| Teaching level | Primary | 199 (54.1%) | 145 (62.5%) | 54 (39.7%) | Chi-square | 0.000115 |
| CPR knowledge | General | 256 (69.6%) | 157 (67.7%) | 99 (72.8%) | Chi-square | 2.86E-09 |
| CPR knowledge | No knowledge | 15 (4.1%) | 13 (5.6%) | 2 (1.5%) | Chi-square | 2.86E-09 |
| CPR knowledge | Only heard | 57 (15.5%) | 51 (22.0%) | 6 (4.4%) | Chi-square | 2.86E-09 |
| CPR knowledge | Very clear | 40 (10.9%) | 11 (4.7%) | 29 (21.3%) | Chi-square | 2.86E-09 |
| Know AED location | No | 161 (43.8%) | 115 (49.6%) | 46 (33.8%) | Chi-square | 1.85E-08 |
| Know AED location | Not sure | 142 (38.6%) | 97 (41.8%) | 45 (33.1%) | Chi-square | 1.85E-08 |
| Know AED location | Yes | 65 (17.7%) | 20 (8.6%) | 45 (33.1%) | Chi-square | 1.85E-08 |
| When CPR is needed | Conscious with chest pain | 7 (1.9%) | 5 (2.2%) | 2 (1.5%) | Chi-square | 6.02E-05 |
| When CPR is needed | Not sure | 45 (12.2%) | 42 (18.1%) | 3 (2.2%) | Chi-square | 6.02E-05 |
| When CPR is needed | Pulse present but dyspnea | 106 (28.8%) | 67 (28.9%) | 39 (28.7%) | Chi-square | 6.02E-05 |
| When CPR is needed | Unresponsive and not breathing (agonal) | 210 (57.1%) | 118 (50.9%) | 92 (67.6%) | Chi-square | 6.02E-05 |
| Golden time | Not sure | 44 (12.0%) | 38 (16.4%) | 6 (4.4%) | Chi-square | 0.004525 |
| Golden time | Over 10 min | 15 (4.1%) | 7 (3.0%) | 8 (5.9%) | Chi-square | 0.004525 |
| Golden time | Within 4 min | 252 (68.5%) | 151 (65.1%) | 101 (74.3%) | Chi-square | 0.004525 |
| Golden time | Within 8 min | 57 (15.5%) | 36 (15.5%) | 21 (15.4%) | Chi-square | 0.004525 |
| Compression location | Chest center | 287 (78.0%) | 167 (72.0%) | 120 (88.2%) | Chi-square | 0.000136 |
| Compression location | Left chest | 45 (12.2%) | 31 (13.4%) | 14 (10.3%) | Chi-square | 0.000136 |
| Compression location | Not sure | 28 (7.6%) | 28 (12.1%) | 0 (0.0%) | Chi-square | 0.000136 |
| Compression location | Right chest | 8 (2.2%) | 6 (2.6%) | 2 (1.5%) | Chi-square | 0.000136 |
| Compression depth | 3-4 cm | 145 (39.4%) | 93 (40.1%) | 52 (38.2%) | Chi-square | 2.01E-09 |
| Compression depth | 5-6 cm | 146 (39.7%) | 72 (31.0%) | 74 (54.4%) | Chi-square | 2.01E-09 |
| Compression depth | >6 cm | 12 (3.3%) | 5 (2.2%) | 7 (5.1%) | Chi-square | 2.01E-09 |
| Compression depth | Not sure | 65 (17.7%) | 62 (26.7%) | 3 (2.2%) | Chi-square | 2.01E-09 |
| Compression rate | 100-120/min | 165 (44.8%) | 93 (40.1%) | 72 (52.9%) | Chi-square | 4.06E-07 |
| Compression rate | 60-100/min | 138 (37.5%) | 79 (34.1%) | 59 (43.4%) | Chi-square | 4.06E-07 |
| Compression rate | >120/min | 8 (2.2%) | 5 (2.2%) | 3 (2.2%) | Chi-square | 4.06E-07 |
| Compression rate | Not sure | 57 (15.5%) | 55 (23.7%) | 2 (1.5%) | Chi-square | 4.06E-07 |
| AED timing | Immediate analysis and shock | 147 (39.9%) | 88 (37.9%) | 59 (43.4%) | Chi-square | 4.45E-09 |
| AED timing | Not sure | 100 (27.2%) | 87 (37.5%) | 13 (9.6%) | Chi-square | 4.45E-09 |
| AED timing | Use after 2 min CPR | 121 (32.9%) | 57 (24.6%) | 64 (47.1%) | Chi-square | 4.45E-09 |
| Worry about emergencies | Never | 25 (6.8%) | 15 (6.5%) | 10 (7.4%) | Chi-square | 0.35383 |
| Worry about emergencies | Often | 88 (23.9%) | 61 (26.3%) | 27 (19.9%) | Chi-square | 0.35383 |
| Worry about emergencies | Seldom | 80 (21.7%) | 53 (22.8%) | 27 (19.9%) | Chi-square | 0.35383 |
| Worry about emergencies | Sometimes | 175 (47.6%) | 103 (44.4%) | 72 (52.9%) | Chi-square | 0.35383 |
| Rescue willingness | Need legal protection | 64 (17.4%) | 43 (18.5%) | 21 (15.4%) | Chi-square | 0.299008 |
| Rescue willingness | Not willing | 16 (4.3%) | 13 (5.6%) | 3 (2.2%) | Chi-square | 0.299008 |
| Rescue willingness | Willing & competent | 102 (27.7%) | 65 (28.0%) | 37 (27.2%) | Chi-square | 0.299008 |
| Rescue willingness | Willing but worried | 186 (50.5%) | 111 (47.8%) | 75 (55.1%) | Chi-square | 0.299008 |
| Skill mastery after training | Almost forgotten | 6 (1.6%) | 0 (0.0%) | 6 (4.4%) | - | |
| Skill mastery after training | Competent independently | 17 (4.6%) | 0 (0.0%) | 17 (12.5%) | - | |
| Skill mastery after training | Know theory only | 29 (7.9%) | 0 (0.0%) | 29 (21.3%) | - | |
| Skill mastery after training | Missing | 0 (0.0%) | 0 (0.0%) | 0 (0.0%) | - | |
| Skill mastery after training | Remember steps but rusty | 84 (22.8%) | 0 (0.0%) | 84 (61.8%) | - | |
| Time since last training | 1-2 years | 22 (6.0%) | 0 (0.0%) | 22 (16.2%) | - | |
| Time since last training | 6-12 months | 34 (9.2%) | 0 (0.0%) | 34 (25.0%) | - | |
| Time since last training | >2 years | 40 (10.9%) | 0 (0.0%) | 40 (29.4%) | - | |
| Time since last training | Missing | 0 (0.0%) | 0 (0.0%) | 0 (0.0%) | - | |
| Time since last training | ≤6 months | 40 (10.9%) | 0 (0.0%) | 40 (29.4%) | - | |
| Volunteer willingness | Depends on time | 133 (36.1%) | 86 (37.1%) | 47 (34.6%) | Chi-square | 0.017143 |
| Volunteer willingness | Not now | 58 (15.8%) | 45 (19.4%) | 13 (9.6%) | Chi-square | 0.017143 |
| Volunteer willingness | Willing | 177 (48.1%) | 101 (43.5%) | 76 (55.9%) | Chi-square | 0.017143 |
| Training necessity | Average | 42 (11.4%) | 31 (13.4%) | 11 (8.1%) | Chi-square | 0.346274 |
| Training necessity | Necessary | 110 (29.9%) | 66 (28.4%) | 44 (32.4%) | Chi-square | 0.346274 |
| Training necessity | Not necessary | 11 (3.0%) | 9 (3.9%) | 2 (1.5%) | Chi-square | 0.346274 |
| Training necessity | Not very necessary | 7 (1.9%) | 4 (1.7%) | 3 (2.2%) | Chi-square | 0.346274 |
| Training necessity | Very necessary | 198 (53.8%) | 122 (52.6%) | 76 (55.9%) | Chi-square | 0.346274 |
| Readiness score | - | 55.7±17.4 | 47.4±13.8 | 69.8±13.3 | Kruskal-Wallis | 3.02E-35 |

**Supplementary Figure 1. Residual diagnostics.**


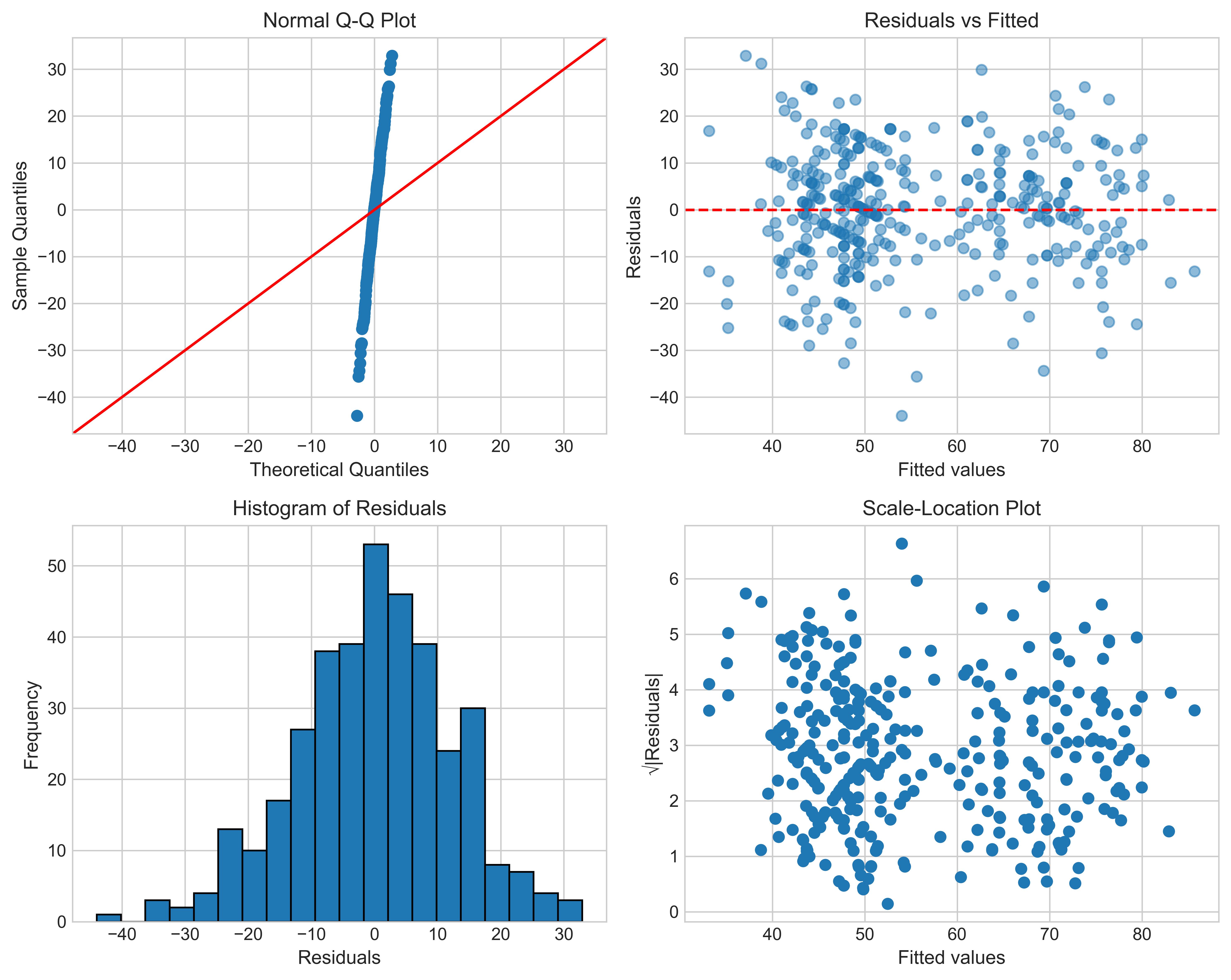


**School Teachers' CPR Knowledge and Skills Survey Questionnaire**

**Part I: Basic Information**

1. Gender: □ Male □ Female
2. Age: □ 18-25 years □ 26-35 years □ 36-45 years □ 46-60 years
3. Ethnicity: □ Han □ Uyghur □ Kyrgyz □ Other: _______
4. Education Level: □ Technical Secondary School □ Junior College □ Bachelor's □ Graduate
5. Teaching Level: □ Elementary School □ Middle School □ High School □ University

**Part II: CPR Awareness and Knowledge Level**

1. What is your level of understanding of CPR?
   □ Very clear (can describe the complete procedure)
   □ Generally understand, but unclear on details
   □ Only heard of the concept
   □ Completely unfamiliar
2. Which of the following situations requires immediate CPR?
   □ Patient is unresponsive, not breathing or not breathing normally (only agonal breathing)
   □ Patient has a pulse but difficulty breathing
   □ Patient is conscious but has chest pain
   □ Uncertain
3. Do you know the locations of AED devices in your school?
   □ Yes (please list specific locations: _______)
   □ No
   □ Uncertain
4. What do you think are the main factors affecting CPR success rate? (Multiple choice)
   □ Rescue time (e.g., starting within the golden 4 minutes)
   □ Standardization of compression depth and rate
   □ Patient's age or underlying conditions
   □ Availability of AED on site
   □ Other: _______

**Part III: CPR Training Experience and Effectiveness**

1. Have you received CPR training?
   □ Yes, attended formal training at hospitals/Red Cross etc.
   □ Yes, self-taught through videos/books
   □ No

(If "No" is selected, please skip to question 13)

1. Time of most recent training:
   □ Within 6 months □ 6 months-1 year □ 1-2 years □ Over 2 years
2. How well did you master the skills after training?
   □ Can perform independently and properly
   □ Remember the steps but rusty on operation
   □ Only understand the theory
   □ Almost forgotten
3. Which training method was most helpful to you? (Multiple choice)
   □ Theoretical explanation □ Model demonstration □ Hands-on practice
   □ VR simulation □ Video cases □ Group discussion □ Other: _______

**Part IV: CPR Procedure Standards Awareness**

1. What is the "golden rescue time" for CPR?
   □ Within 4 minutes □ Within 8 minutes □ Over 10 minutes □ Uncertain
2. What is the correct position for adult chest compressions?
   □ Center of chest (midpoint of line between nipples)
   □ Left chest
   □ Right chest
   □ Uncertain
3. What should be the depth of chest compressions?
   □ 5-6 centimeters □ 3-4 centimeters □ Over 6 centimeters □ Uncertain
4. What should be the rate of chest compressions?
   □ 100-120 times/minute □ 60-100 times/minute □ Over 120 times □ Uncertain
5. What are the correct steps for rescue breathing? (Multiple choice)
   □ Open airway (head tilt-chin lift maneuver)
   □ Pinch patient's nostrils closed
   □ Seal mouth over patient's mouth and blow
   □ Each breath 1 second, observe chest rise
   □ Uncertain
6. When should AED be used after arrival?
   □ Analyze rhythm and defibrillate immediately
   □ Use after completing 2 minutes of CPR
   □ Uncertain

**Part V: Rescue Willingness and Training Needs**

1. Are you worried about encountering emergency situations requiring CPR?
   □ Often worried □ Occasionally worried □ Rarely worried □ Never worried
2. If someone needs CPR, what is your willingness to provide rescue?
   □ Willing and able to perform properly
   □ Willing but worried about making mistakes
   □ Would only provide rescue with legal protection
   □ Unwilling to participate
3. In what format do you hope the school will conduct CPR training? (Multiple choice)
   □ Regular offline hands-on classes
   □ Expert lectures + certification assessment
   □ Online courses + simulation practice
   □ Community self-training equipment
   □ First aid knowledge competition
   □ Other: _______
4. Are you willing to join the school's CPR volunteer promotion team?
   □ Willing □ Depends on schedule □ Not considering for now
5. How necessary do you think it is for teachers to receive CPR training?
   □ Very necessary □ Necessary □ Neutral □ Not very necessary □ Unnecessary
